# The Effects of the Fraction Isolated from Iranian *Buthotus shach* Scorpion Venom on Synaptic Plasticity, Learning, Memory, and Seizure Susceptibility

**DOI:** 10.5812/ijpr-138273

**Published:** 2023-10-30

**Authors:** Elmira Heidarli, Hossein Vatanpour, Nafiseh Nasri Nasrabadi, Maha Soltani, Saeed Tahmasebi, Mehrdad Faizi

**Affiliations:** 1 Department of Pharmacology and Toxicology, School of Pharmacy, Shahid Beheshti University of Medical Sciences, Tehran, Iran; 2 Pharmaceutical Sciences Research Centre, School of Pharmacy, Shahid Beheshti University of Medical Sciences, Tehran, Iran; 3 Department of Cognitive Science, Science and Research Branch, Islamic Azad University Tehran, Tehran, Iran

**Keywords:** Epilepsy, Scorpion, Memory, Learning, Synaptic, Plasticity, Field, Recording, Acute, Seizure.

## Abstract

Epilepsy, as a neurological disease, can be defined as frequent seizure attacks. Further, it affects many other aspects of patients’ mental activities, such as learning and memory. Scorpion venoms have gained notice as compounds with potential antiepileptic properties. Among them, *Buthotus schach* (BS) is one of the Iranian scorpions studied by Aboutorabi et al., who fractionated, characterized, and tested this compound using electrophysiological techniques in brain slices (patch-clamp recording). In the present study, the fraction obtained from gel electrophoresis was investigated through behavioral and electrophysiological assays. At first, ventricular cannulation was performed in rats, and then the active fraction (i.e., F3), carbamazepine, and the vehicle were microinjected into the brain before seizure induction by the subcutaneous (SC) injection of pentylenetetrazol (PTZ). Seizure behaviors were scaled according to Racine stages. Memory and learning were evaluated using the Y-maze and passive avoidance tests. Other groups entered evoked field potential recording after microinjection and seizure induction. Population spike (PS) and field excitatory postsynaptic potential (fEPSP) were measured. The F3 fraction could prevent the fifth stage and postpone the third stage of seizure compared to the control (carbamazepine) group. There was no significant improvement in memory and learning in the group treated with the F3 fraction. Also, PS amplitude and fEPSP slope increased significantly, and long-term potentiation was successfully formed after the high-frequency stimulation of the performant pathway. Our results support the antiepileptic effects of the F3 fraction of BS venom, evidenced by behavioral and electrophysiological studies. However, the effects of this fraction on memory and learning were not in the same direction, suggesting the involvement of two different pathways.

## 1. Background

Epilepsy is one of the most common neurological impairments around the world. Statistics show an incidence rate of 10% in the general population (1). Frequent seizure attacks aside, epilepsy can have profound negative effects on cognition, consciousness, and motor function (2). Current treatments for epilepsy are mainly divided into two groups: Pharmaceutical therapy and surgery. Both of these therapeutic approaches may cause serious side effects, and they may not be able to completely control seizure attacks. Also, current medications merely alleviate epileptic symptoms but have no impact on epileptogenesis (3). Another remarkable point is that epilepsy medicines have a wide range of interference with other medications and even foods.

Long-term potentiation (LTP) is defined as the purposeful reorganization or regeneration of neurons in response to persistent stimuli in order to change neuronal function and structure (or even both) to strengthen neuronal reaction to the stimulus (4). The hippocampus region has an enormous role in LTP formation in the brain, and the Dentate gyrus region of the hippocampus is one of the most important players in this event (5). Long-term potentiation is known to have a substantial role in regulating brain functions, such as memory and learning, and also in the development of diseases like epilepsy and depression (4, 6). The fact that epilepsy can cause memory and learning problems has been widely studied (7-10), and both of these conditions are believed to be rooted in the same origin and follow similar mechanisms. Therefore, it is crucial to more deeply explore this potential link.

Natural compounds are gaining attention as potential treatments for seizures and epilepsy. Researchers have proved the anti-seizure potency of compounds like flavonoids, terpenoids, and alkaloids (11). A large number of polypeptides with anti-seizure properties have been extracted and purified from animals, including wasps, bees, spiders, and scorpions (12, 13). Scorpion envenomation is a lethal situation that can lead to death in a significant number of victims. There are controversial reports on the clinical outcomes and applications of scorpion envenomation. One of the symptoms in people stung by scorpions is acute seizure. On the other hand, there is growing interest in purifying the fractions of scorpion venom with anti-seizure properties, leading to promising achievements (14). For instance, one of the neurotoxins derived from the scorpion *Buthotus martensi* Karsch could prevent seizure in rat models of PTZ (15). In another study, a peptide from scorpion *Heterometrus spinifer* successfully reduced the severity of seizure in mice models of PTZ-induced seizure (16).

Scorpion venoms are mixtures of different peptides, including long-chain peptides with modulating effects on sodium channels and short-chain peptides with blocking effects on potassium channels. *Buthotus schach* (BS) is a scorpion belonging to the Buthidae family with a limited distribution in some parts of Iran. The most recognized symptoms following BS envenomation are arrhythmia, respiratory depression, convulsion, and cardiac arrest (17).

## 2. Objectives

Regarding the fact that scorpion venoms affect ion channels and interfere with cellular functions, we investigated if BS venom could modulate the function of ion channels in Wistar rats.

## 3. Methods

### 3.1. Experiment 1

#### 3.1.1. Effects of F3 Fraction on Susceptibility to Seizure, Learning, and Memory

*Buthotus schach* crude venom was provided by the Department of Poisonous Animals, Razi Vaccine and Serum Research Institute, Karaj, Iran. The venom was obtained by electrically stimulating the scorpion telson and then was lyophilized using a freeze dryer (18). Venom fractions were separated by gel filtration as described in a previous study. According to Aboutorabi et al., the F3 fraction, compared to other fractions, could decrease sodium flow in patch clamp recording. It has been confirmed that sodium flow has a significant role in the occurrence and progression of seizures, so its blocking may promote anti-seizure effects. Regarding the above-mentioned, we decided to divulge the potential anti-seizure effects of this fraction more deeply (17).

#### 3.1.2. Animals

Eighteen male Wistar rats in the weight range of 250 - 300 gr were randomly divided into three groups (n = 6 per group). The animals were obtained from the Pasteur Institute (Tehran, Iran), had free access to water and pellet food, and were kept in standard condition (12/12 h light-dark cycle, temperature of 22 ± 2ºC, and 40% humidity). All experiments were carried out according to the guidelines of the Ethical Committee of Shahid Beheshti University of Medical Sciences, and the code of ethics was IR.SBMU.PHARMACY.REC.1402.038.

The animals were anesthetized by ketamine (100 mg/kg) and xylazine (10 mg/kg) and fixed on the stereotaxic apparatus. An incision was made on the skin, and extra tissue was carefully removed from the target area. After the spots and midline became visible, the left ventricle of the brain was marked and drilled. A stainless-steel guide cannula was placed in the whole, according to Paxinos and Watson's atlas. Dentistry cement was used to fix the cannula and close the wound. After a 5- to 7-day recovery period, the rats were injected by the target fraction through the intra-cerebroventricular (i.c.v) route. Control animals received normal saline; carbamazepine-injected rats were regarded as positive controls (400 µg/rat). The last group received the active fraction of BS venom (F3, 10 µg/kg). Thirty minutes after the i.c.v injection, 60 mg/kg of PTZ was subcutaneously injected into the back in all groups. Seizure behaviors were observed for 30 minutes and scaled in reference to the Racine seizure grading system. The Y-maze and passive avoidance tests were performed after seizure induction to assess the effects of seizure on learning and memory.

In the Y-maze test, animals were placed in the central part of a 3-compartment maze, and the number and order of entrance in each arm were recorded for 10 minutes. The alternation percentage was calculated and compared between the groups (19).

A passive avoidance test was performed right after the Y-maze. Animals were placed in the bright compartment and moved freely to get familiar with the environment. Then, the gate between the two compartments was opened, and animals entered the dark compartment out of curiosity. When the test object came to the dark compartment, a low-frequency unpleasant stimulus (current = 0.2 mA, frequency = 50 Hz, duration = 5 s) was applied to the animal’s feet. It made a memory to prevent further entrance. On the second day of the experiment, animals were placed in the bright compartment, and the gate was opened. The cut-off time was set as 120 seconds, and not entering the dark compartment during this time was considered as passing the test; otherwise, the animal failed the test. The experiment was repeated 3 times after the first adaptation test (20).

### 3.2. Experiment 2

#### 3.2.1. Electrophysiological Studies

Evoked-field potential (e-LFP) animals were divided into 4 groups (6 rats in each group); stereotaxic surgery and the i.c.v injection of the active fraction were performed as described in the previous section (i.e., experiment 1, behavioral studies). After 30 min, PTZ was subcutaneously injected into the animals (60 mg/kg), which were anesthetized by the intraperitoneal injection of Urethane (1.5 g/kg). Thirty minutes after seizure induction, animals’ heads were fixed on the apparatus using ear bars. Covering cement was removed; the skull was cleaned, and the two target points (dentate gyrus and perforant pathway) were marked, where holes were drilled into the skull. Electrode coordinates were applied for the performant pathway (anteroposterior = −8.1 mm; mediolateral = ±4.3 mm; dorsoventral = 3 – 3.3 mm) and dentate gyrus in the left hemisphere (anteroposterior = −3.8 mm; mediolateral = ±2.4 mm; dorsoventral = 2.7 – 3.2 mm). Two electrodes made of double-strand stainless steel wire were placed above the marked regions and lowered slowly to reach the accurate point under the skull surface according to the spike shape, and this process was closely monitored on a screen. A stability period of 10 - 20 min was given to the recording cells. After that, the I/O curve was plotted, and the test stimulus current was obtained from 60% of the maximum response. Signals were amplified (100×) and filtered (1 to 3 kHz bandpass) using a differential amplifier. Baseline recording was performed for 15 minutes (one stimulus with a frequency of 100 mHz was induced every minute, and the spike was recorded). Then, LTP was induced according to the high-frequency stimulation protocol (10 trains of 20 pulses at 400 Hz with 80% of maximum intensity every 10 seconds). In the final step, the dentate gyrus area was monitored for 60 minutes. It was expected to see a 25% acceleration in two measuring parameters, including population spike (PS) amplitude and excitatory postsynaptic potential (EPSP) (i.e., the slope of the line connecting the first and second peaks of the record). Rats with incorrect electrode positioning were excluded. Recording and data analysis were performed by e-probe and e-lab setup (21, 22).

## 4. Results

### 4.1. Experiment 1

**Figure 1.**
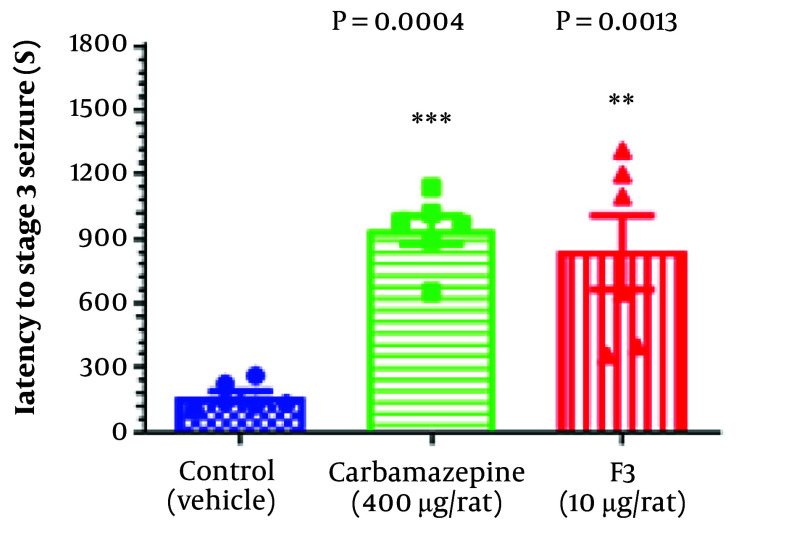
The effect of F3 fraction on latency to stage 3 PTZ-induced acute seizure in rats. Six animals were used in each group. One-way ANOVA was used for multiple comparisons between groups, followed by Tukey’s post-hoc test. Mean ± SD was reported.

In behavioral studies, statistical analysis showed that the administration of the F3 fraction of BS venom increased latency to the stage 3 seizure according to the Racine seizure scale. Remarkably, the effectiveness of the F3 fraction was considerably close to that of carbamazepine (an approved epilepsy medicine), both of which could postpone the occurrence of stage 3 PTZ-induced seizure. Figure 1 depicts the impact of the F3 fraction on latency to stage 3 seizure in comparison with other groups.

**Figure 2.**
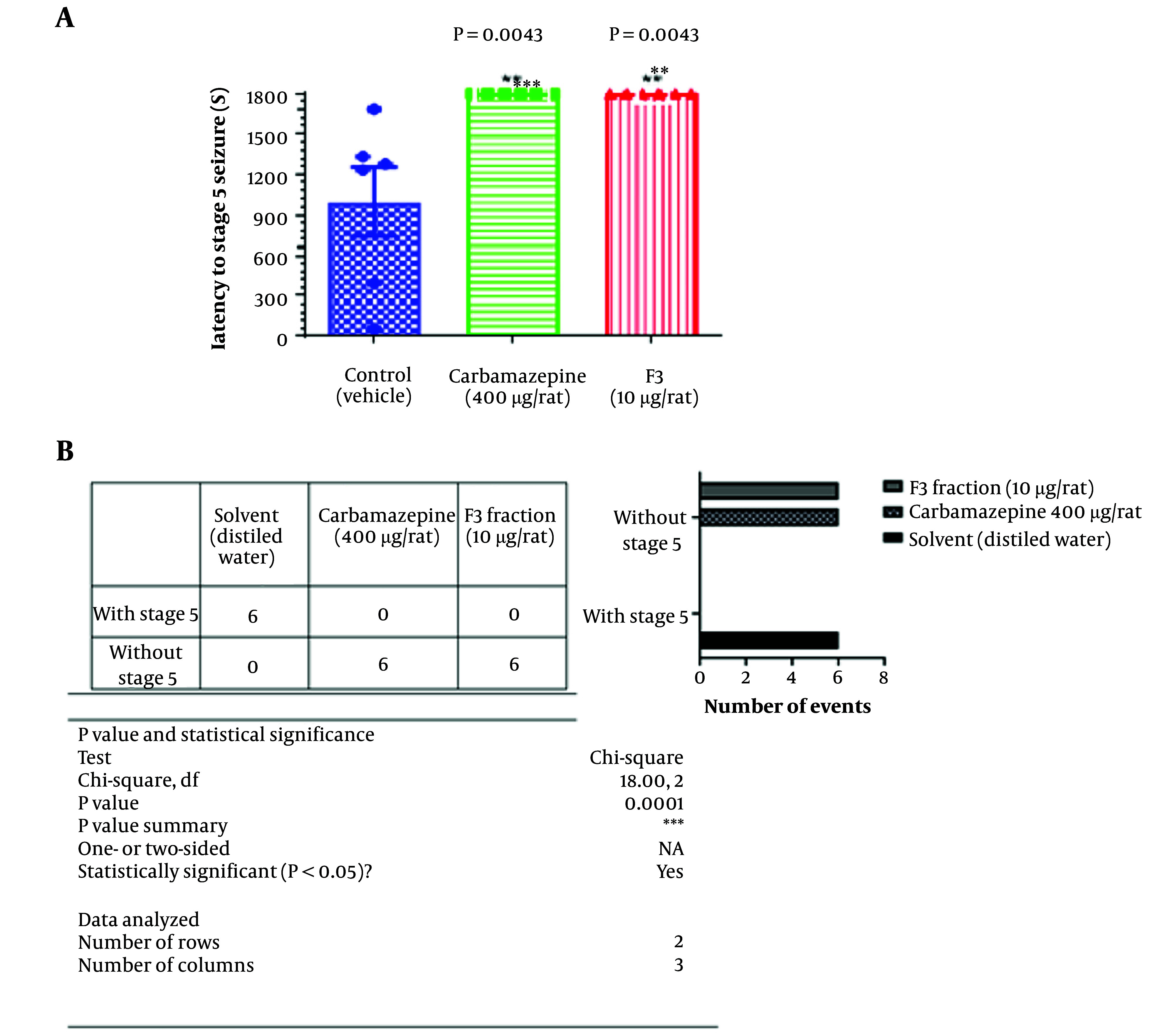
The effect of F3 fraction microinjection on stage 5 seizure latency of PTZ-induced acute seizure in rats. Six animals were used in each group. One-way ANOVA was used to analyze this set of data and the recommended test for multiple comparisons between groups was Tukey. Mean with SD was reported.

The effects of the F3 fraction on latency to stage 5 (tonic-clonic) seizure that is shown in Figure 2. The F3 fraction could completely prevent the occurrence of tonic-clonic seizures.

In another comparison we used the contingency table and compared the number of stage 5 seizure between groups by using chi-square test. As it is shown in part B of the Figure 2, F3 fraction and Carbamazepine completely prevented the stage 5 occurrence when, all the animals in vehicle received group, showed the highest level of Racine scale (stage 5. Tonic- clonic seizure).

### 4.2. Y-maze and Passive Avoidance Tests

**Figure 3.**
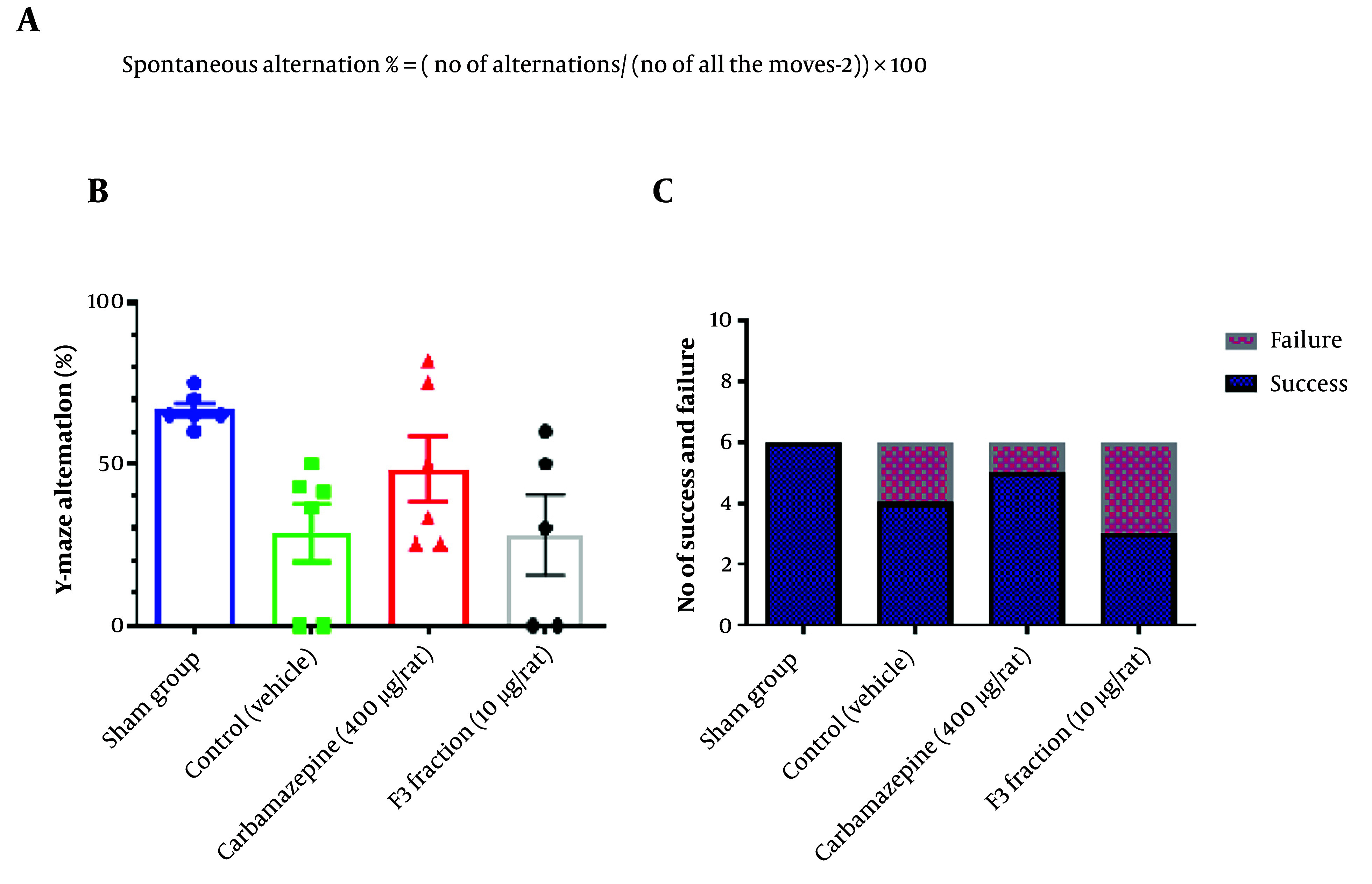
Assessment of compound i.c.v administration on memory and learning after PTZ-induced seizure. A) The equation has been used to calculate the percentage of alternation in Y- maze test. B) Carbamazepine improved the alternation percent in animals but not in a significant way (Number in each group= 6, One-way ANOVA was used to analyze this set of data, and the recommended test for multiple comparisons between groups was Tukey. Mean with SD, Significant difference p value < or = 0.05 (95% confidence interval)) - C) Carbamazepine showed a better seizure control effect than other groups but it was not significant. (Number in each group= 6, Chi-square test was used to analyze this set of data.

In the Y-maze test, we calculated the alternation percentage. The number and order of animals’ entries to the arms were recorded. A consecutive rotation between the two arms was considered an alternation. The number and percentage of alternations were calculated using the equation shown in Figure 3A. The results showed that there was no significant difference between the study groups, meaning that the F3 fraction could not improve memory and learning in animals (Figure 3B). 

The passive avoidance test was repeated three times for each animal at a cut-off time of 120 seconds. No entry to the dark compartment after 120 seconds was considered passed; otherwise, the test result was regarded as failed. In this test, no significant difference was observed in the number of animals entering the dark compartment between the rats treated with the F3 fraction and other groups. Figure 3 shows the results of these two tests. The results of the passive avoidance test have been shown in Figure 3C. 

### 4.3. Experiment 2

**Figure 4.**
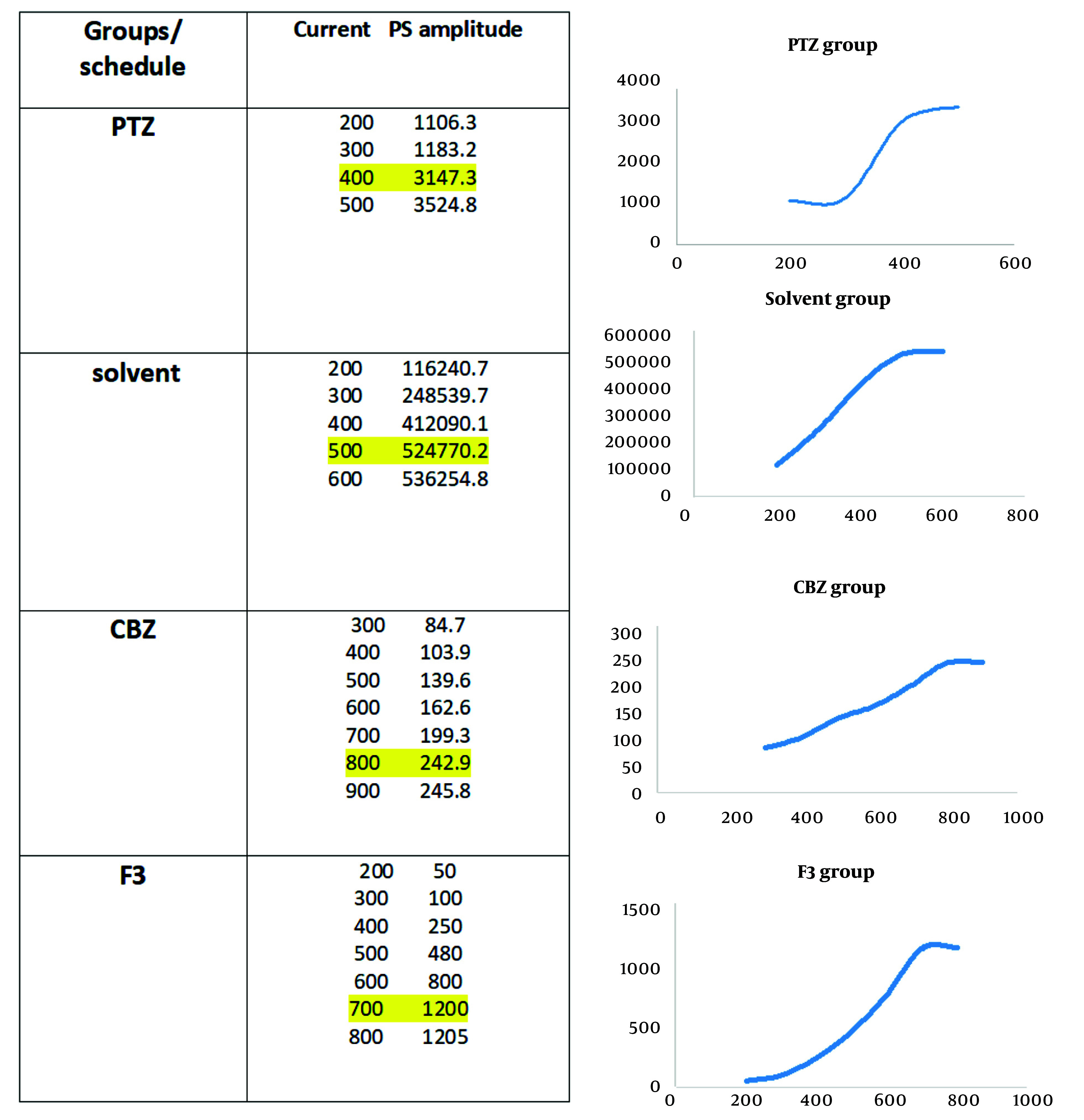
The method to access the intensity. Input stimuli were given to the neuronal population and the PS amplitude was recorded; when it reached a steady state, 60% of maximum intensity was considered as the goal stimulus. Highlighted points are maximum responses that no significant increase was seen after them.

In electrophysiological studies, recording was performed in the dentate gyrus area, and data were obtained before and after LTP induction according to the high-frequency stimulation (HFS) protocol. Between- and within-group analyses (Figure 4A) indicated some traces in field potential recording before and after HFS induction in the study groups. Also, the Input/Output curve (I/O curve) was provided (part B in the very same figure).

**Figure 5.**
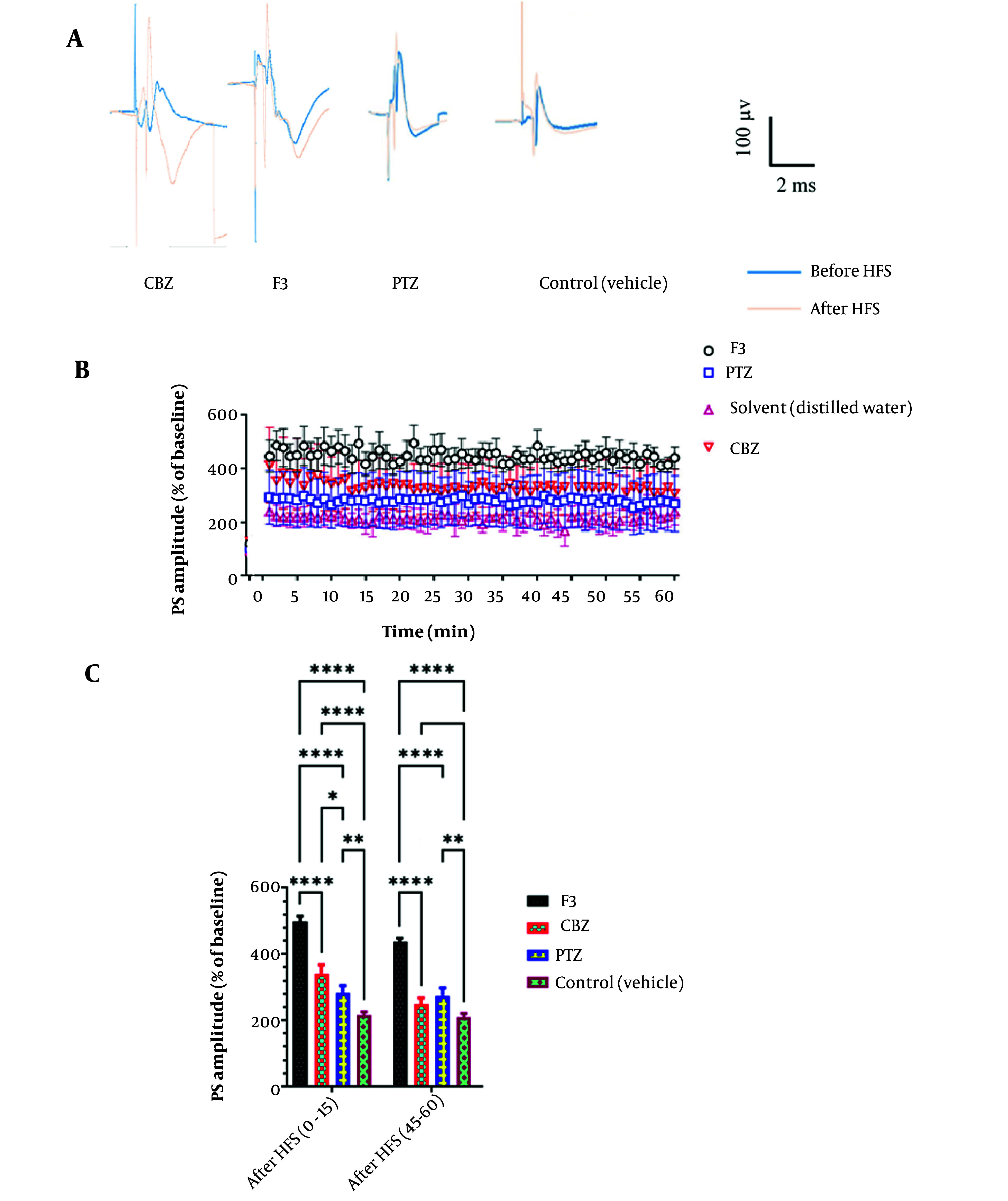
A, Examples of recordings from the DG region before and after LTP induction in different groups. B, Effect of F3 fraction of BS venom on PS amplitude in local field potential recording. According to the results of two-way ANOVA analysis, F3 causes a significant increase in the PS amplitude factor at almost all time points compared to the control group (n = 8). C, B, Comparison between PS amplitude of different groups in certain time intervals. As shown in the graph, two-way ANOVA analysis shows a significant difference between the group receiving F3 and the other groups in both time periods. Also, in the first fifteen minutes after LTP induction, the group receiving CBZ significantly increased the PS amplitude parameter.

Postsynaptic activities were recorded by the extracellular field potential recording technique. Baseline activities and post-LTP behaviors were compared at different times (Figure 5). Statistical analysis (two-way ANOVA) demonstrated a significant impact on F3 fraction [F (3, 1036) = 121.1, P < 0.0001]. There was also a significant difference between the groups at different time points [(74, 1036) = 6.747, P < 0.0001]. Administration of the F3 fraction significantly increased PS amplitude in comparison with the control group (vehicle) in almost all time points. Rats in other groups (CBZ and PTZ) showed no significant changes in PS-LTP amplitude. So, it can be concluded that the F3 fraction of BS could affect the action potential pathway and neuronal firing by modulating the activity of ion channels.

**Figure 6.**
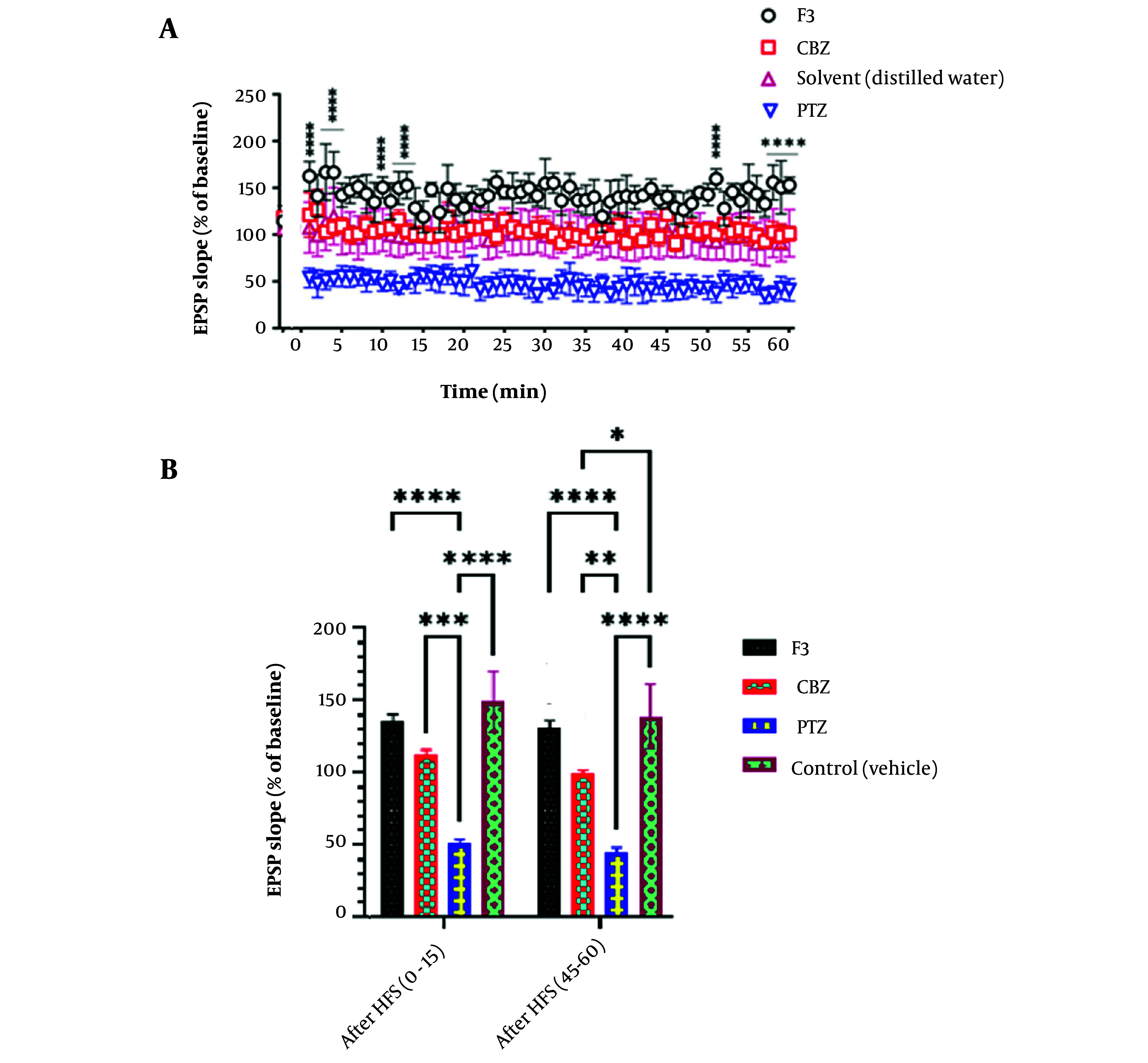
The effect of BS venom F3 fraction on EPSP slope in local field potential recording. A, According to the results of two-way ANOVA analysis, F3 causes a significant increase in the EPSP slope factor at almost all time points compared to the control group. (n = 8). B, In the first and last fifteen minutes of recording, the F3 group had a significant increase in the EPSP slope compared to the groups receiving PTZ and CBZ. Also, the increase in the solvent receiving group was significant compared to the PTZ and CBZ receiving groups.

In another measurement, fEPSP factor was recorded and compared between groups (Figure 6). Also, in fEPSP slope, F3 group, shows a significant increase after HFS induction in all time points (Figure 5, part A) and in the first and last 15 minutes after HFS (Figure 5, part B)

## 5. Discussion

Our results showed that the active fraction of BS venom could affect PTZ-induced seizure severity and the latency of each stage. As it has been proven in previous research that was performed by Aboutorabi et al., this fraction had a different impact on ion channels in comparison with the whole venom and other fractions in patch clamp technique studies which was claimed for its anti-epileptic properties (17).

The study was followed by two behavioral tests to assess the impact of the F3 fraction on memory and learning impairment which is caused by PTZ-induced seizure (23, 24). It is known that epilepsy and seizure could lead from an LTP pattern to epileptogenesis and this can disturb the pathway of memory formation (25, 26). Y-maze and passive avoidance tests were performed to discover the effect of active compounds on the pattern of short-term and long-term memory function after seizure (27), respectively. Our results did not support this hypothesis that seizure suppression correlates with memory improvement after administration of the F3 compound because there was no significant difference between the F3 received group and others. It could be a hint that F3 may interact with different pathways and acts through other mechanisms than the one that participates in memory and learning patterns.

LTP is one of the most important synaptic plasticity forms, in both physiological and pathological events in the brain. LTP happens in response to repetitive stimulus which leads to potentiating the connections and functions of neurons in a specific way (4). As it has been pointed out previously, the hippocampus, and specifically the dentate gyrus region, are mostly responsible for LTP formation (28). Field potential recording is one of the electrophysiological tools that is widely used to study brain mechanisms. Several studies used field potential recording to track LTP and in most of them, the target area was the dentate gyrus of the hippocampus (21, 22, 29, 30). In this study, the field potential technique was used to complete the results of the behavioral section. fEPSP slope and PS amplitude were the recorded factors. PS amplitude is an indicator of activation of a population of neurons and ion movements to generate action potentials (31). According to our results, the F3 fraction of BS has an increasing effect on PS amplitude. Previous research has found three major functions for different peptides in scorpion venoms (32). The first group includes peptides affecting sodium current that interact with open/closed gates (33). The second group modulates potassium ions by the pore-blocking mechanism and the last group modulates calcium current through Ryanodine channels (34, 35). It seems that the F3 fraction has an effect on neuronal firing after high-frequency stimulation which could be originated from sodium pathway interactions. PS amplitude rise following F3 fraction application may be the first steps of memory and learning formation in a convulsive brain which couldn’t be proved in our results in the behavioral section. According to previous studies, NMDA receptors and their binding proteins have a great role in LTP and LTD formation in the hippocampus. For instance, in the study of Migaud et al., a mutation that led to lowering the amount of PSD-95, an NMDA receptor binding protein, caused an imbalance between LTP and LTD pattern and shifted the activity toward LTP. Behavioral studies showed that learning and memory are a result of a balanced state of LTP and LTD and increasing the stimulus frequency to over-express LTP level can impair LTD formation and have a negative consequence on memory and learning (36). In another study by Gu et al., the Fmr2 gene was knocked out and the same result was achieved (37). In our study, F3 may have the same effect on LTP-LTD pattern and although LTP has potentiated in recordings, we could not see any positive impacts on memory and learning.

Repetitive firing can provide a saturated environment in synaptic space that prevents LTP formation. Seizure enforces neuronal networks to function at a frequent strong level that saturates synapses and prevents robust LTP formation in the DG following high-frequency stimulation of the perforant pathway. Our results indicate that, the group that received F3 before seizure induction showed higher synaptic flexibility mirrored by stronger LTP expression in contrast to epileptic rats treated with vehicle. Seemingly, F3 regulates the tone and frequency of neuronal activity keeps it in a balanced state, and provides free space for synaptic potentiation and LTP formation.

Field excitatory postsynaptic potential measures the excitatory drive to the neurons (38). After analysis, we found F3 could also increase the fEPSP slope in a significant way. This data was predictable because, PS is the indicator of LTP induction in axons as a result of the current generated in dendrites of the neurons, and EPSP shows the activity in dendrites.

As has been described in the previous paragraph, F3 modulates synaptic activity and prevents the unbridled firing of neurons. As shown in the related graph, as a result of controlling effect of the F3, fEPSP has been increased after HFS induction. However, several neurons are active in this situation, they can’t reach the highest level of activation. Also, it could be related to the fall of driving force to enter sodium ions into the cell membrane, because of depolarization of resting membrane potential after HFS induction and disability of membrane to reach the previous resting potential and sodium homeostasis.

### 5.1. Conclusions

In conclusion, our results show that F3 fraction isolated from BS scorpion venom could enhance neuronal connections and plasticity through LTP induction as well as seizure prevention in PTZ injected rats. However, it could not make significant changes on memory and learning following seizure induction.
